# Open-Circuit Voltage Models Should Be Thermodynamically Consistent

**DOI:** 10.1021/acs.jpclett.3c03129

**Published:** 2024-01-24

**Authors:** Archie Mingze Yao, Venkatasubramanian Viswanathan

**Affiliations:** † Department of Mechanical Engineering, Carnegie Mellon University, Pittsburgh, Pennsylvania 15213, United States; ‡ Department of Aerospace Engineering, University of Michigan, Ann Arbor, Michigan 48109, United States

## Abstract

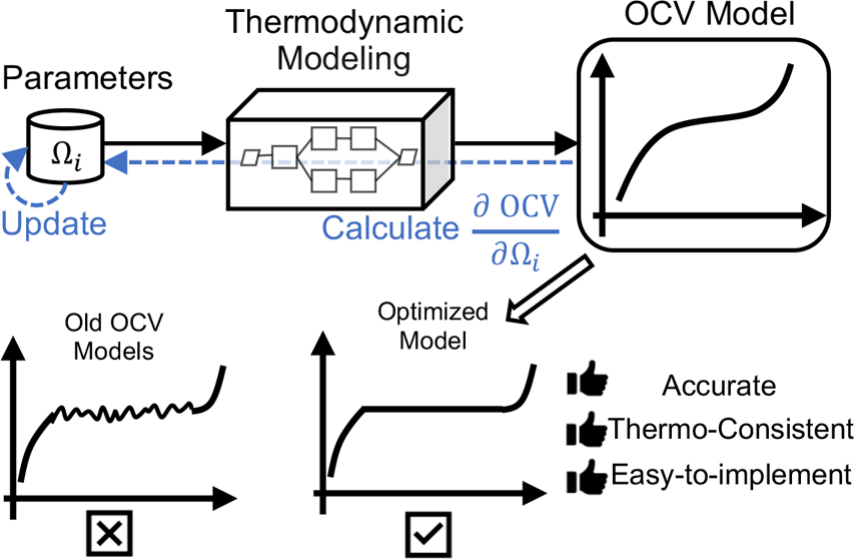

Open-circuit voltage (OCV) models as a function of state-of-charge (SOC) are fundamental to modeling the performance of batteries. The second law of thermodynamics enforces that the OCV should be monotonic with respect to the SOC. In this Perspective, we first review some of the currently popular empirical OCV fitting methods which compromise thermodynamics for the flexibility of empirical fitting. We propose a simple thermodynamically consistent OCV model enabled by differentiable thermodynamic modeling, which obeys the second law of thermodynamics. We cast the common-tangent condition derived from the second law of thermodynamics as a fixed-point solving problem and use implicit function theorem to enable efficient gradient-based parameters optimization. We demonstrate this on the OCV of 12 popular electrode materials, and this is integrated with open-source battery modeling software PyBaMM. We perform pseudo-2D discharge simulations to show the seamless integration of the above OCV models with battery modeling software. Given the simplicity of integration and implementation, we believe that thermodynamically consistent OCV models should be a requirement for future Li-ion battery models.

*The open-circuit voltage (OCV) is the steady-state voltage when no current flows through a battery and is a fundamental thermodynamic property of the electrodes.*(1,2) Getting accurate OCV as a function of state-of-charge (SOC) is critical in understanding physical phenomena^3^ and battery performance under various operating conditions^4^ and for battery management systems.^5^ OCV of electrode materials of lithium (Li)-ion batteries can be derived according to thermodynamics. The intercalation reaction of lithium (Li) ion into an electrode material of a Li-ion battery (i.e., electrode material), HM, is given by
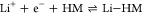
1For example, when the host material is FePO_4_, the intercalation reaction is Li^+^ + e^–^ + FePO_4_ ⇌ LiFePO_4_. Enforcing chemical equilibrium of the intercalation reaction leads to μ_Li^+^_ + μ_e^–^_ + μ_HM_ = μ_Li–HM_, where μ_X_ stands for the chemical potential of species X. Rearranging the equation, we get μ_e^–^_ = μ_Li–HM_ – μ_HM_ – μ_Li_. The chemical potential of Li–HM, i.e., μ_Li–HM_, can be derived from the expression of molar Gibbs free energy of Li–HM, i.e., *g*_Li–HM_, as . The lithiation process of the host material can be viewed as mixing Li^+^ with vacancy sites inside HM. According to the thermodynamics of mixing,^6^ the molar Gibbs free energy of Li–HM can be decomposed into three contributions, the pure substances contribution, the ideal mixing contribution, and the excess enthalpy contribution,^7^ as *g*_Li–HM_ = *g*_pure_ + *g*_ideal_^mix^ + *g*_excess_^mix^, where *x* is the mole fraction of Li, 0 ≤ *x* ≤ 1, *g*_pure_ = *xμ*_Li–HM_^o^ + (1 – *x*)μ_HM_^o^, and *g*_ideal_^mix^ = *RT*(*x* log *x* + (1 – *x*) log(1 – *x*)), respectively. *g*_excess_^mix^ is the excess Gibbs free energy due to interaction (i.e., nonideal effect) between species. Therefore, we have the chemical potential of electron as , where μ^o^ = μ_Li–HM_^o^ – μ_HM_^o^ – μ_Li_^o^ is the reference chemical potential and is a constant. Since μ_e^–^_ = −*FU*, we have
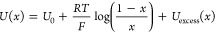
2where *U*_0_ is the reference voltage, *R* is the perfect gas constant, *T* is the temperature, *F* is the Faraday constant, *x* is the Li filling fraction to the electrode material, and *U*_excess_(*x*) is the excess enthalpy contribution as a function of *x*. The second law of thermodynamics enforces that in a closed isothermal, isobaric ensemble, the second-order derivative of the Gibbs free energy of component *i*, i.e., *G*_*i*_, and the amount of component *i*, i.e., *N*_*i*_, satisfies , so that the system is stable.^8^*Equivalently, in a battery, this implies that the chemical potential of the electron, or equivalently the OCV, should be a monotonic function of the SOC.*

Popular OCV models include various empirical fittings with different function forms and splines as summarized in Table 1 and Pillai et al.^19^ Although they are good at minimizing fitting error, most of these OCV models are not supported by thermodynamics, sacrificing underlying physics for the flexibility of empirical fitting. For example, many models^13,14,16,17,20,21^ applied exponential functions to model the divergence of OCV near SOC = 0 and SOC = 1. However, such behavior of the OCV arises from the ideal mixing term in eq 2 and should be therefore modeled with logarithmic function. Spline-based OCV models work well in some conditions, but such OCV models cannot give correct function gradients and therefore cannot guarantee other important thermodynamic quantities that depends on the OCV, for example, entropic heat  and molar volume . A popular thermodynamic-based practice of OCV modeling is to follow the above-described thermodynamic derivation of the OCV and expand the excess enthalpy contribution *g*_excess_^mix^ in various ways, e.g., Redlich–Kister (R-K) polynomials.^18^ For example, Plett,^1^ in the textbook for battery modeling, gives an analytical expression of OCV with respect to the Li filling fraction (*x*) of lithium iron phosphate (LFP) and many other electrode materials based on R-K polynomials with an empirical skew factor. However, the fitted OCV models violate the second law conditions stated above. Karthikeyan et al.^18^ has used various thermodynamic expansions for the chemical potential to fit a wide variety of OCV curves, two of which violate the second law condition (see Supporting Information, Table 1). One downside of violating the monotonic condition is that SOC estimation based on the OCV value is infeasible because the mapping from the OCV value to the SOC value is not one-to-one. Also, since the OCV model violates thermodynamics, the thermodynamic properties derived from the OCV may not be valid. Some OCV models violating the second law of thermodynamics are summarized in Table 2 and Figure 1. More examples of OCV models that violate the second law of thermodynamics can be found in Supporting Information. The general issue of violation of monotonicity was first recognized by Clerk-Maxwell in 1875^22^ and addressed by the proposal of phase coexistence determined through the Clerk-Maxwell construction method. Although Clerk-Maxwell construction is a convenient graphical method, programmatic implementation is difficult as it involves integrals evaluation.^22^ An equivalent and easier to implement method is the common tangent construction for phase coexistence.^8^ A simple approach to ensuring that the OCV models are monotonic would be to combine them with the common tangent method. However, a practical difficulty is that the fitting procedure must integrate the OCV models with the common tangent method, which makes the optimization of the parameters in the OCV models hard due to the iterative solving process of the phase boundary. Differentiable programming enables taking gradients through entire programs;^23^ the parameters can be optimized with gradient-based methods which thus provides an approach to address this problem.

**Table 1:** Summary of OCV vs SOC Models

Ref	General Expression	Description
(9−11)	*U* = ∑_*i*=0_^*n*^*a*_*i*_*x*^*i*^	*n*^*th*^-order polynomial model
(12−14)	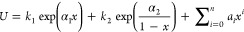	Double exponential functions model
	or *U* = *k*_1_ exp(*α*_1_*x*) + *k*_2_ exp(*α*_2_(1 – *x*)) + ∑_*i*=0_^*n*^*a*_*i*_*x*^*i*^	
(9), (10), (15)	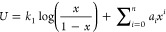	Logarithmic function model
(16)	*U* = *k*_1_ exp(α_1_*x*) + ∑_*i*=0_^*n*^*a*_*i*_*x*^*i*^	Exponential function model
(17)	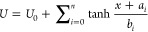	Hyperbolic tangent functions model
(1)	See eqs 7 and 8	Skewed R-K model
(18)	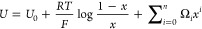	*n*-parameter Margules model
(18)	See eqs 2 and 3	R-K model

**Table 2:** OCV Models in Existing Literature That Violate the Monotonic Condition and Thus the Second Law of Thermodynamics

Ref	Description	SOC Region
(18)	Two-parameter Margules model	[0.40, 0.80]
(1)	Skewed R-K model	[0.08, 0.16]
(9)	8^*th*^-order polynomial model	[0.78, 0.93]
(12)	Double exponential function model	[0.80, 0.90]
(10)	6^*th*^-order polynomial model	[0.10, 0.24]
(11)	4^*th*^-order polynomial model	[0.25, 0.55]

**Figure 1:**
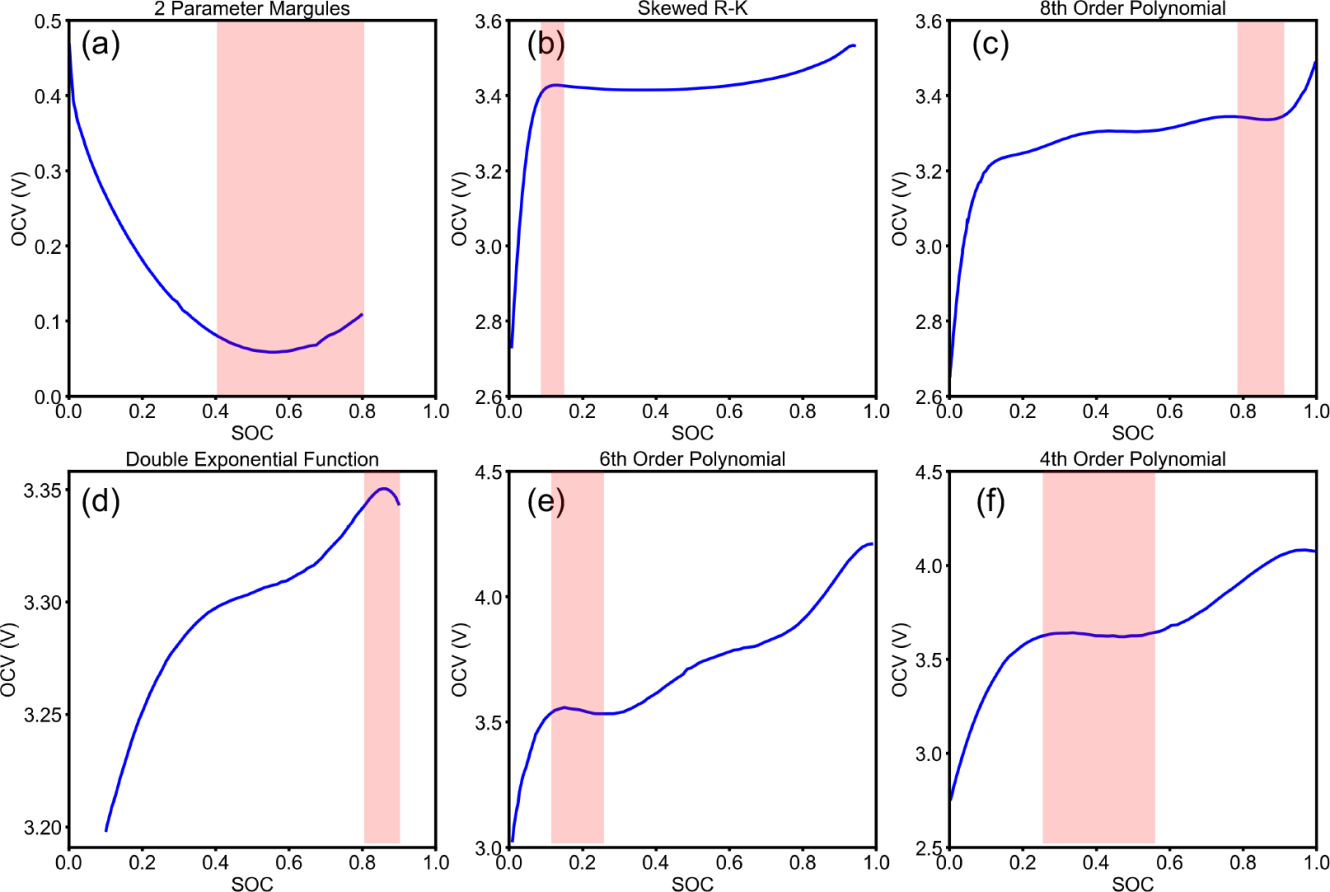
OCV models in existing literature that violate the monotonic condition and thus the second law of thermodynamics. Nonmonotonic regions of these models are highlighted in red rectangles. Data digitized with WebPlotDigitizer.^24^ (a) Two-parameter Margules OCV model for mesocarbon microbeads (MCMB).^18^ (b) Skewed R-K model for LFP.^1^ (c) 8^*th*^-order polynomial model for LFP.^9^ (d) Double exponential function model for a LFP|graphite battery.^12^ (e) 6^*th*^-order polynomial model for a NMC|graphite battery.^10^ (f) 4^*th*^-order polynomial model for NMC.^11^

Here, we propose a thermodynamically consistent OCV fitting method with phase equilibrium calculations performed using gradient-based optimization enabled by differentiable programming. We demonstrate the power of this approach by showing the OCV of LFP can be fit with a polynomial basis of only third order, while obtaining any realistic fits without this approach requires >10 degree polynomial. We then apply the thermodynamically consistent model for 12 electrode materials and construct a thermodynamically consistent OCV function database. We integrate the developed thermodynamically consistent OCV functions with open-source battery modeling software PyBaMM^25^ and demonstrate pseudo-2D (P2D) discharge simulations of LFP|Graphite Cylindrical 18650 cell and NMC111|Graphite pouch cell at C/20, C/2, 1C, and 2C. The simulated discharging profiles reproduce the corresponding experimental results well. We believe that this approach should become the standard for the modeling of the OCV in Li-ion battery models.

Our OCV model is expressed in eq 2. To parametrize *U*_excess_(*x*) in eq 2, we apply R-K polynomials to expand excess Gibbs free energy as *g*_excess_^mix^ = *x*(1 – *x*)∑_*i*=0_^*N*^Ω_*i*_(1 – 2*x*)^*i*^, where *x* is the mole fraction of Li, 0 ≤ *x* ≤ 1. Ω_*i*_ is the *i^th^*-order R-K coefficients. Therefore, we have the chemical potential of electron as
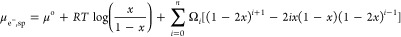
3where μ^o^ = μ_Li–HM_^o^ – μ_HM_^o^ – μ_Li_^o^ is the reference and sp stands for single phase. The above derivation assumes that the electrode active material undergoes no phase separation. However, many electrode materials undergo phase separation during cycling. For example, LFP undergoes phase separation into a Li-rich phase and a Li-poor phase in a wide range of lithium filling fraction.^26^ In this phase separation region, the common tangent construction as shown in eq 4 needs to be applied in order to obtain the coexistence chemical potential μ_coex_.
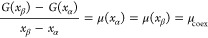
4

The phase separation region, [*x*_α_, *x*_β_] is first roughly decided by the convex hull algorithm which gives an initial guess and then solving common tangent condition shown in eq 4 to refine the initial guess. Given the coexistence chemical potential, the chemical potential of electron equals to the expression in eq 3 when *x* < *x*_α_ or *x* > *x*_β_, and equals to μ_coex_ when *x*_α_ ≤ *x* ≤ *x*_β_. Finally, the fitted OCV is expressed as
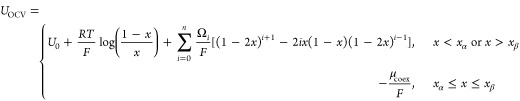
5where *F* is the Faraday constant and *n* is the order of the R-K expansion.

To optimize the R-K coefficients Ω_*i*_ in eq 3, we construct a loss function between the above calculated chemical potential of electron from eq 5 and the real chemical potential of electron, backpropagating through the loss function to get the sensitivity of loss function with respect to the R-K parameters and update the R-K parameters with gradient-based optimization algorithms like Adam.^27^ Because μ_e^–^_ = −*FU*_OCV_, we can calculate the real chemical potential of the electron, μ_e^–^_^true^, from the measured OCV value. The loss function is defined as *L* = ∑_*i*_(μ_e^–^_^true^(*x*_*i*_) – μ_e^–^_(*x*_*i*_))^2^, where μ_e^–^_(*x*_*i*_) is the chemical potential at filling fraction of Li at *x* = *x*_*i*_ given by eq 5 and μ_e^–^_^true^(*x*_*i*_) is the corresponding true value calculated from the measured OCV.

If *x*_*i*_ is outside the miscibility gap, calculating  (i.e., backpropagation) is an easy task for any automatic differentiation framework. However, calculating  when *x*_*i*_ is located inside the miscibility gap is nontrivial, because μ_e^–^,*i*_ is calculated from the common tangent construction. Guan^28^ calculated  by writing the convex hull algorithm and the common tangent solving process in JAX^29^ so that all operations are tracked. Calculating , where x* = [*x*_α_, *x*_β_], therefore requires backpropagating through the Newton–Raphson solver used to solve the common tangent condition and the convex hull algorithm, which is memory and time inefficient. Here, inspired by the backpropagation implementation of the deep equilibrium model,^30^ we reframe the common tangent solving problem as a fixed point solving problem by rewriting the common tangent condition shown in eq 4 as
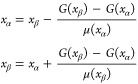
6With x* = [*x*_α_, *x*_β_] defined as the fixed point, *f* as the operation on the right-hand side of eq 6, θ as the R-K parameters Ω_*i*_, and the reference chemical potential μ^o^, the common tangent condition is now rewritten as a fixed-point solving problem, x* = *f*(x*, θ). Backpropagating through a fixed point equation can be done via differentiating the fixed-point equation directly through the implicit function theorem as . In this way, the sensitivity of fixed-point x* with respect to parameters θ is calculated directly instead of tracking the iterative fixed-point solving process. The above backpropagation scheme avoids tracking the fixed point solver and getting speed improvement compared with the implementation of Guan.^28^ The workflow of the above OCV fitting algorithm (referred to as the “thermodynamically consistent OCV model” below) can be found in Figure 2.

**Figure 2:**
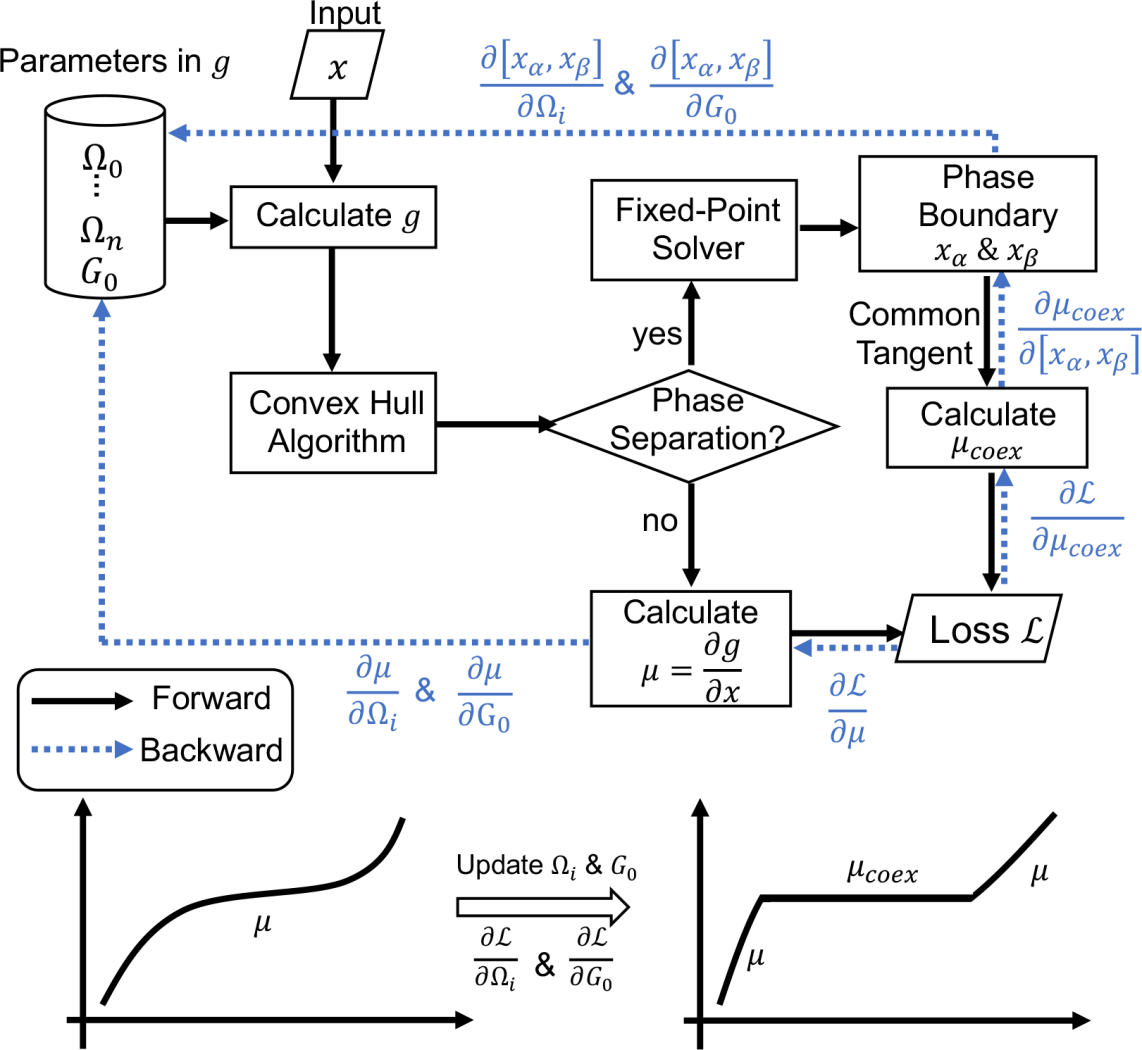
Schematic workflow for calculating the OCV with differentiable thermodynamic modeling. Starting from Li filling fraction *x* and the thermodynamic parameters Ω_0_, ..., Ω_*n*_, the molar Gibbs free energy, *G*_0_, of electron *g* can be calculated. The convex hull algorithm is then applied to detect the concave region, therefore deciding whether there is a phase separation region or not. If no phase separation happens, the chemical potential is the partial derivative of *g* and can be calculated with automatic differentiation. If, however, there is a concave region (i.e., phase separation region), the fixed-point solver is applied to solve the rewritten common tangent problem shown in eq 6 to get the phase boundary [*x*_α_, *x*_β_]. The coexisting chemical potential μ_coex_ can thus be calculated with eq 4. Finally, the loss function is constructed as the mean squared error between predicted chemical potential and measured chemical potential. Because the common tangent condition is rewritten as a fixed-point solving problem, backpropagation can be wisely implemented instead of tracking through the fixed-point solver.

First, we apply the above OCV fitting method to the discharge OCV of LFP, which is poised to be one of the dominant cathodes for electric vehicle applications.^31^ The excess enthalpy contribution term is expanded with R-K polynomials to the third order, i.e., *n* = 3 in eq 5. The experimental discharge OCV profile of LFP at 25 °C is digitized with WebPlotDigitizer^24^ from Dreyer et al.^32^ The resulting OCV model can be found in Figure 3 as the red dotted line. We compare the optimized OCV curve with three other thermodynamic-based models: the first one is the regular R-K method, expressed as eq 2 with *U*_excess_(*x*) expanded with R-K polynomials and shown as a dashed green line in Figure 3, where the values of *U*_0_ and Ω_*i*_ are fitted directly to the experimental results. To minimize the RMSE of up to 0.0001 V while at the same time keeping minimal the number of parameters in the model, the excess Gibbs free energy is expanded with 30^*th*^-order R-K polynomials, i.e., *n* = 30. The second model is the Monotonic R-K shown as the dotted blue line in Figure 3 where, in addition to the regular R-K model, monotony of the fitted OCV curve is enforced by adding the constraints,^33^*U*(*x*_*i*_) ≤ *U*(*x*_*i*+1_), where *x*_*i*_ < *x*_*i*+1_. The constraints ensure the fitted OCV curve is monotonic with respect to SOC within the fitted range, thus respecting the second law of thermodynamics. To minimize the RMSE of up to 0.0001 V while at the same time keeping minimal the number of parameters in the model, the excess Gibbs free energy is expanded with 51^*th*^-order R-K polynomials, i.e., *n* = 51. The last model is the skewed R-K model from Plett et al.^1^ shown as the dotted magenta line in Figure 3 and expressed as eqs 7 and 8,
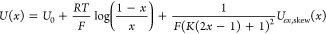
7
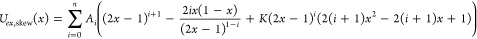
8where the values of *A*_*i*_, *U*_0_, *K*, and *n* = 15 come from Plett et al.^1^Table 3 summarizes the *R*^2^ score and root mean squared error (RMSE) of the four models. The thermodynamically consistent OCV model proposed in this work has significantly lower RMSE and higher *R*^2^ score compared with the monotonic R-K model, which also respects the second law of thermodynamics. The regular R-K model has a lower RMSE because it has more parameters than the thermodynamically consistent OCV model but fails to respect the second law of thermodynamics as it is not monotonic, as shown in Figure 3(c). The skewed R-K model has the same issue, as shown in Figure 3(b). Also, as the regular R-K model adopts high-order R-K polynomial expansion of excess enthalpy and is fitted within the SOC range given by the experimental data, the predicted OCV value decreases drastically for the SOC outside the fitted range, as shown in 3(d). However, the model proposed in this work does not have such unphysical behavior, as the excess enthalpy is expanded to only third order; therefore, no overfitting is observed. More detailed explanations can be found in the Supporting Information. It is worth mentioning that the high-order expansion of excess enthalpy in the regular R-K model is necessary in order to fit the plateau region in the discharging OCV profile, as essentially the regular R-K model is trying to fit a constant (i.e., the plateau region) with a polynomial expansion.

**Figure 3:**
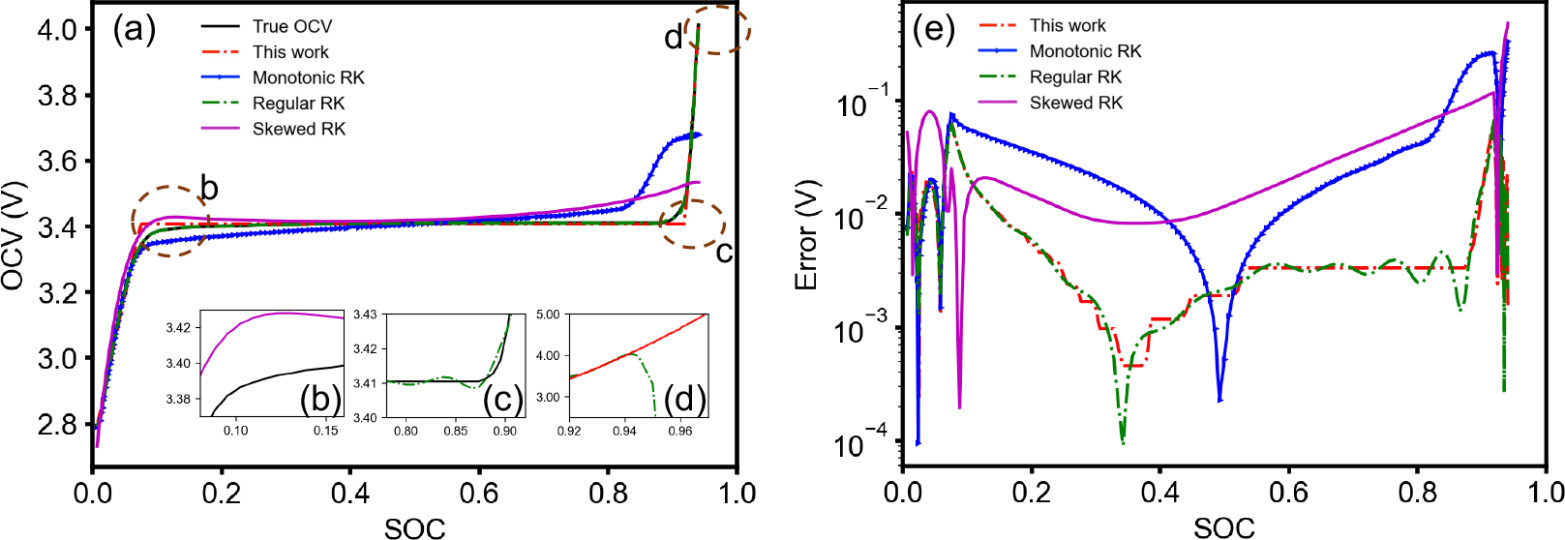
(a) Fitted discharge OCV of LFP curves by different methods. Skewed R-K fit uses the parameters given in Plett et al.^1^ (b) and (c) Zoom-in for Skewed R-K and Regular R-K, respectively, showing that they are not monotonic, thus violating the second law of thermodynamics. (d) The behavior of Regular R-K is unphysical outside the fitted region; it decreases drastically. Because low-order R-K expansion is used, this work gives a very reasonable extrapolation. (e) Absolute error plot of all four models. The oscillation of absolute error of the regular R-K model is caused by the nonmonotonicity shown in (c).

**Table 3:** *R*^2^ Score, Root Mean Squared Error (RMSE) of the Models in Figure 3

Model Name	This work	Monotonic R-K	Regular R-K	Skewed R-K
*R*^2^	0.9954	0.8467	0.9998	0.7444
RMSE (V)	0.0145	0.0841	0.0028	0.1085
Monotonic?	Yes	Yes	No	No

As a demonstration of the transferability of the thermodynamically consistent OCV fitting method, the method is applied to the OCV profile of 11 popular cathode and anode materials (other than LFP) as well. For cathode materials, we fit the OCV profile of Li_*x*_FeSiO_4_^32^ (discharge profile), LiCoO_2_ (LCO),^34^ LiMnPO_4_ (LMP),^35^ LiMn_0.5_Fe_0.5_PO_4_ (LMFP),^35^ LiMn_2_O_4_ (LMO),^36^ LiNi_0.847_Co_0.127_Al_0.026_O_2_ (NCA),^37^ LiNi_0.4_Co_0.6_O_2_ (NCO),^38^ and LiNi_0.8_Mn_0.1_Co_0.1_O_2_ (NMC).^39^ For anode materials, we fit the OCV profile of graphite,^40^ silicon^41^ (Si, assuming the final lithiation product is Li_15_Si_4_), and Li_4/3_Ti_5/3_O_4_.^42^ The results are summarized in Figure 4. For comparison, we also fit the monotonic R-K models with exactly the same amount of total parameters that the corresponding thermodynamically consistent OCV model has. The 12 fitted thermodynamically consistent OCV models shown in Figure 4 are provided as PyBaMM OCV functions. Most thermodynamically consistent OCV models have RMSE of around 0.01 V compared with the corresponding experimental value. The fitted LMFP model, as shown in Figure 4(e) has a RMSE of 0.045 V, which is the largest one among the RMSE value of all the 12 models. RMSE values of all models are reported in the Supporting Information. The error of the thermodynamically consistent OCV model mainly comes from the left boundary of the miscibility gap, because the thermodynamically consistent OCV model causes a discontinuity of  at the boundaries due to the common tangent construction, while the experimental value changes smoothly. Such unsmoothness around the boundary of the miscibility gap is observed in all fitted thermodynamically consistent OCV models where the miscibility gap presents, and the experimental value always changes smoothly. A potential approach to resolve the unsmoothness issue is to use cubic splines at the boundaries of the miscibility gap so that both the OCV value and the first-order derivative of the OCV with respect to the SOC are continuous. A more detailed explanation of this approach can be found in the Supporting Information. It is worth mentioning that the thermodynamically consistent OCV model captures the phase-separation regions well. For example, the fitted OCV model for LFP, as shown in Figure 4(a), contains a miscibility gap between Li filling fraction *x* = 0.08 and *x* = 0.92, which is very close to the experimental results.^26^ The fitted model for graphite, as shown in Figure 4(j), contains two miscibility gaps, i.e., between Li filling fraction *x* = 0.28 and *x* = 0.43 and between Li filling fraction *x* = 0.56 and *x* = 0.82, which agrees with the analysis of Gallagher et al.^40^ that there is phase coexistence of Li_*x*_C_32_ and Li_*x*_C_12_ and phase coexistence of Li_*x*_C_12_ and Li_*x*_C_6_. For those materials without a plateau region, i.e., NCO shown in Figure 4(g), NCA shown in Figure 4(h), and NMC shown in Figure 4(i), the thermodynamically consistent OCV model falls back to the regular R-K model. It is worth mentioning that the monotonicity of the fitted OCV model is still enforced inherently, even if the output model has the same expression of a regular R-K model. This is because the nonmonotonic regions within the fitted model will be detected by a convex hull algorithm which detects the concave, i.e., nonmonotonic, regions in the Gibbs free energy landscape and then eliminated by common tangent construction during the fitting process, as shown in Figure 2.

**Figure 4:**
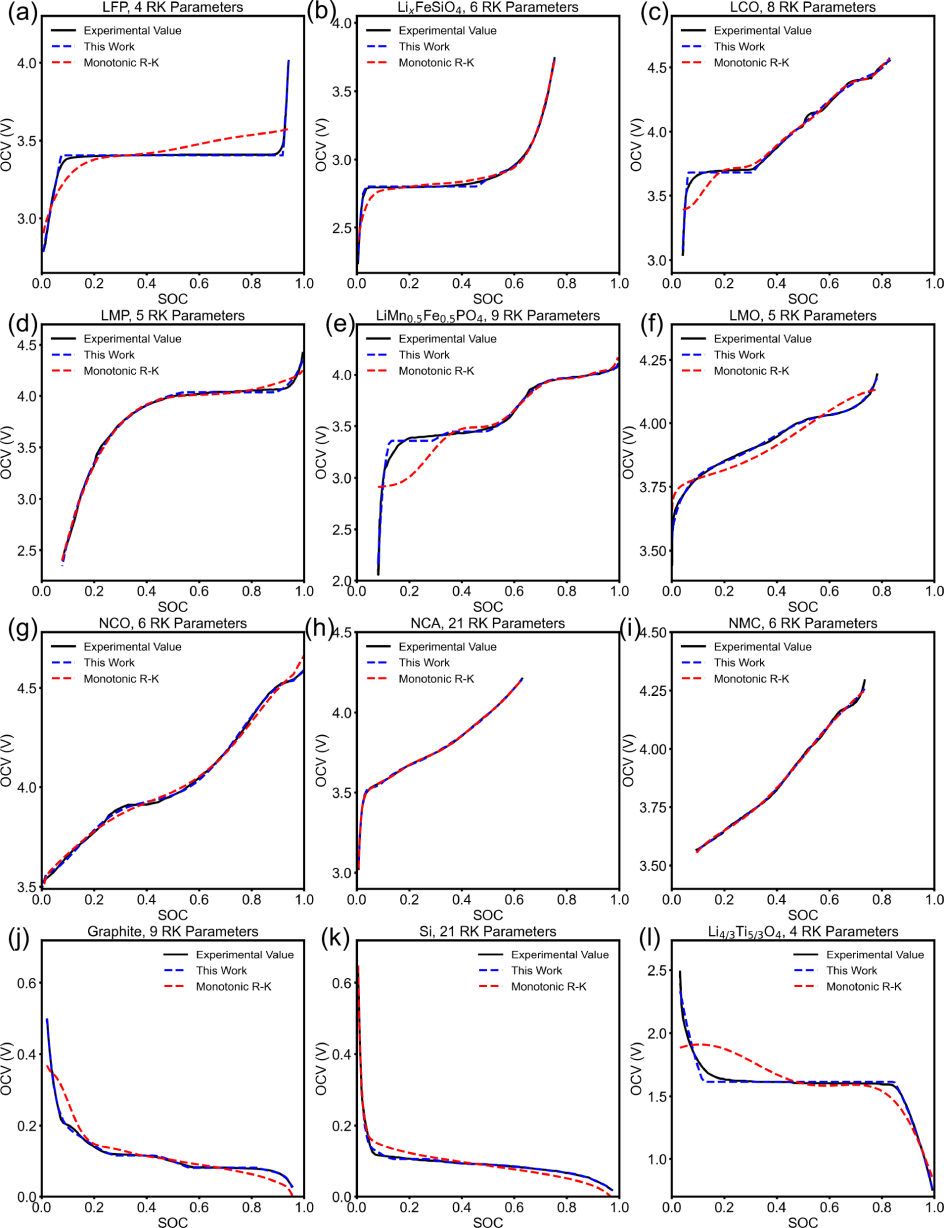
Fitted thermodynamically consistent OCV models (in blue lines, referred to as “this work” in legends) and monotonic R-K OCV models (in red lines) of (a) LFP (discharge)^32^ using 4 R-K parameters, (b) Li_*x*_FeSiO_4_ (discharge)^32^ using 6 R-K parameters, (c) LiCoO_2_ (LCO)^34^ using 8 R-K parameters, (d) LiMnPO_4_ (LMP)^35^ using 5 R-K parameters, (e) LiMn_0.5_Fe_0.5_PO_4_^35^ using 7 R-K parameters, (f) LiMn_2_O_4_ (LMO)^36^ using 5 R-K parameters, (g) LiNi_0.4_Co_0.6_O_2_ (NCO)^38^ using 6 R-K parameters, (h) LiNi_0.847_Co_0.127_Al_0.026_O_2_ (NCA)^37^ using 21 R-K parameters, (i) LiNi_0.8_Mn_0.1_Co_0.1_O_2_ (NMC)^39^ using 6 R-K parameters, (j) graphite^40^ using 7 R-K parameters, (k) silicon (Si)^41^ using 21 R-K parameters, and (l) Li_4/3_TI_5/3_O_4_^42^ using 4 R-K parameters. The experimentally measured OCV values are plotted in black solid lines; the fitted OCV models using the method stated above are plotted in blue dashed lines.

Now comes the issue of whether such a model can be easily integrated into battery models to make this a routine part of P2D battery modeling. Although the training process of the OCV model involves potentially time-consuming algorithms including convex hull algorithms for concave region detection, finding the phase boundary which evolves from the iterative solving process of fixed-point, etc., the actual implementation can be relatively simple. Because the phase boundary has been obtained from the fixed-point solving process during training, we can now implement this OCV model as a piecewise function shown in eq 5, where the OCV value is constant inside miscibility gaps and calculated with the fitted expression outside miscibility gaps. Therefore, there is no need to incorporate any of the thermodynamic modeling algorithms shown in Figure 2 into P2D battery modeling. As a demonstration, we now incorporate the piecewise OCV function for LFP (as shown in Figure 4(a)) and graphite (as shown in Figure 4(j)) into PyBaMM,^25^ perform two sets of P2D^43^ discharge simulations at different C rates at room temperature, one set for a 2 Ah LFP|Graphite cylindrical 18650 cell and the other set for a 12.5 Ah NMC111|Graphite pouch cell (34 single cells connected in parallel), and compare the two sets of P2D simulation results with publicly available experimental results.^44^ The parameters used in the P2D simulations are parametrized by About:Energy Limited and are publicly available.^44^ Details and results of simulations can be found in the Supporting Information. In summary, the simulated discharging profiles of both cells at all four C rates match the experimental results well.

In conclusion, we have introduced a thermodynamically consistent OCV model enabled by differentiable programming, which resolves a long-standing issue in battery modeling for phase-transforming electrodes. We demonstrate that the thermodynamically consistent OCV model proposed here outperforms the existing thermodynamics-based OCV modeling approach for the canonical Li-ion battery cathode, LFP. We then apply the thermodynamically consistent OCV model for 12 commercially relevant electrode materials and construct, to the best of our knowledge, the largest up-to-date PyBaMM thermodynamically consistent OCV model OCV function database. Lastly, we showed the seamless integration of the thermodynamically consistent OCV model with PyBaMM and demonstrated P2D discharge simulations of LFP|Graphite Cylindrical 18650 cell and NMC111|Graphite pouch cell at various C rates. The simulated discharge profiles reproduce the corresponding experimental results well. Given the ubiquity of phase transformation in Li-ion anodes and cathodes, we believe that this approach should become the norm for fitting of OCV models in the future given similar computational complexity, cost, and ease of integration into pseudo-2D models.

## Data Availability

The implementation of the above stated algorithm, the PyBaMM simulation input script, and results can be found at https://github.com/BattModels/Diffthermo_OCV_paper. The twelve OCV models shown in Figure 4 are provided as PyBaMM OCV functions and are publicly available at https://github.com/BattModels/Diffthermo_OCV_paper/tree/main/pybamm_OCV_functions.

## Supporting Information Available

P2D discharge simulations, extrapolation of regular R-K model and thermodynamically consistent model, adding splines at boundaries of miscibility gaps, comparison between thermodynamically consistent OCV model and monotonic R-K model, table of RMSE of the thermodynamically consistent OCV model and monotonic R-K OCV model, OCV models in existing literature that violates second law of thermodynamics (PDF)
