# Supplementary material for: Open-Circuit Voltage Models Should Be Thermodynamically Consistent
Source: J Phys Chem Lett. 2024 Jan 24;15(4):1143–51. doi: 10.1021/acs.jpclett.3c03129 (PMC10839898; doi:10.1021/acs.jpclett.3c03129)
Supplement: Supplementary file 1 — jz3c03129_si_001.pdf [file jz3c03129_si_001.pdf]

Supporting Information for Open-Circuit Voltage Models Should Be Thermodynamically Consistent

Archie Mingze Yao[†] and Venkatasubramanian Viswanathan[*,†,‡]

†*Department of Mechanical Engineering, Carnegie Mellon University, Pittsburgh*

‡*Department of Aerospace Engineering, University of Michigan*

E-mail: venkvis@cmu.edu

P2D Discharge Simulations of 2 Ah Graphite—LFP Cylindrical 18650 cell and 12.5 Ah NMC111—Graphite Pouch Cell

As discussed in main text, we incorporate the piecewise OCV function for LFP (as shown in Figure 4(a) in the main text) and graphite (as shown in Figure 4(j) in the main text) into PyBaMM,[1] and perform two sets of P2D[2] discharge simulations at different C rates at room temperature, one set for a 2 Ah LFP—Graphite cylindrical 18650 cell, the other set for a 12.5 Ah NMC111—Graphite pouch cell (34 single cells connected in parallel), and compare the two sets of P2D simulation results with publicly available experimental results.[3] The parameters used in the P2D simulations are parameterized by About:Energy Limited and are publicly available.[3] For PyBaMM discharge simulations of the LFP—Graphite cylindrical 18650 cell, the OCV function of LFP is the thermodyanmically-consistent OCV model shown

in Figure 4(a), initial SOC is set so that the maximum OCV of the cell is 3.65V,[3] discharge time is set according to the corresponding discharging time of experiments,[3] i.e. when the cell voltage reaches 2.0V. For PyBaMM discharge simulations for the NMC111—Graphite pouch cell, the OCV function of graphite is the thermodyanmically-consistent OCV model shown in Figure 4(j), initial SOC is set so that the maximum OCV of the cell is 4.2V,[3] discharge time is set according to the corresponding discharging time of experiments,[3] i.e. when the cell voltage reaches 2.7V. The built-in Casadi solver[4] is applied to solve all the simulations.

Figure 1 and 2 shows the PyBaMM simulated results of LFP—Graphite Cylindrical 18650 Cell and the NMC111—Graphite pouch cell discharged at C/20, C/2, 1C and 2C respectively. The simulated discharging profiles of both cells at all four C rates matches the experimental results well at most regions. For nearly fully discharged regions of the LFP—Graphite cylindrical 18650 cell, discrepancy between PyBaMM simulations and experimental results is observed. The error may come from the fact that the LFP OCV is fitted according to the experimental results from Dreyer et. al.,[5] where the measured specific capacity of LFP cathode is reported as 160 mAh/g instead of the theoretical capacity of LFP (169 mAh/g). The difference between the measured capacity and theoretical capacity is because the measured LFP cathode contains carbon black and Teflon binder,[5] which bring down the measured capacity of the cathode. Fitting LFP OCV function with higher-quality data might help minimizing the error between the P2D simulation results and the experimental results.

Extrapolation of regular R-K model and thermodynamically consistent model

As mentioned in the main manuscript, the regular R-K model behaves unphysically when extrapolating, while the model proposed in this work does not have unphysical behaviour. To

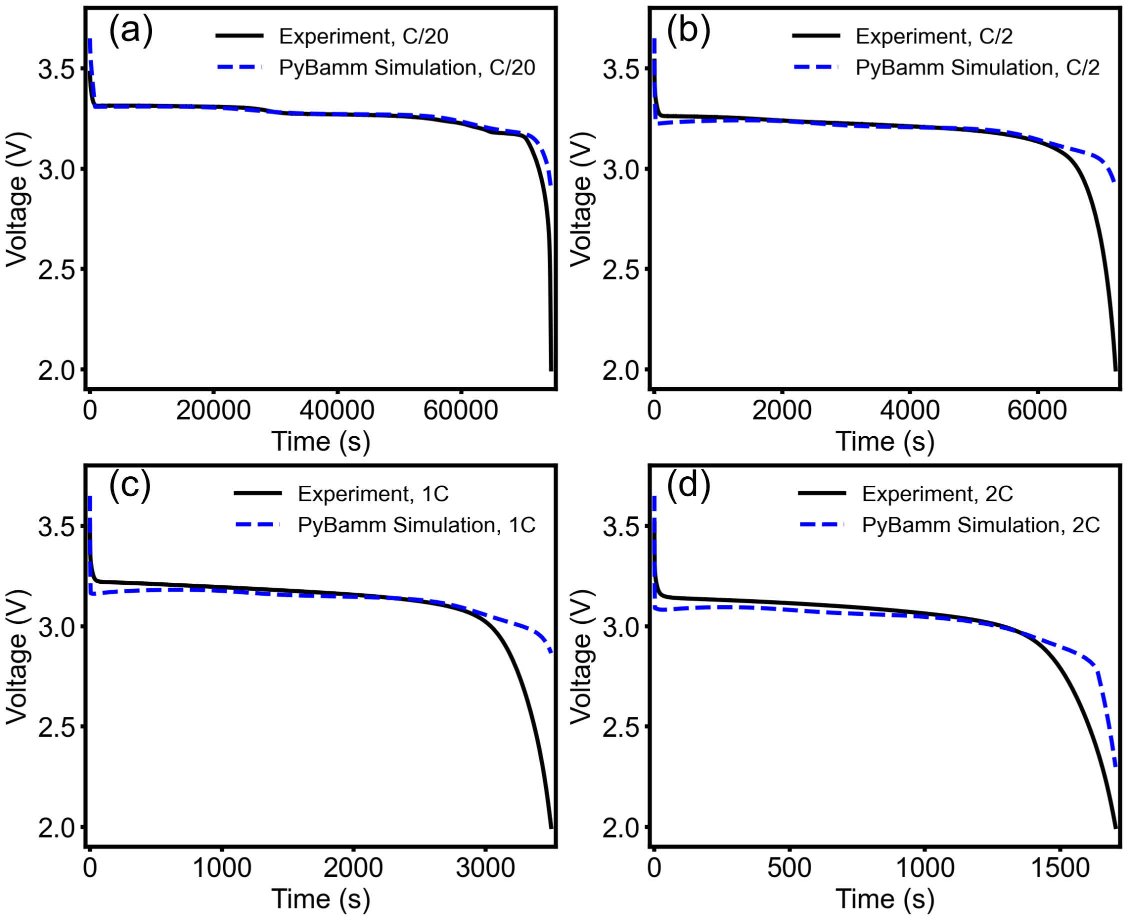

Figure 1: P2D discharge simulations (in blue dash lines) of a 2 Ah Graphite—LFP Cylindrical 18650 cell at room temperature at (a) C/20, (b) C/2, (c) 1C and (d) 2C, and the corresponding experimental results (in black solid line). For all four simulations, the OCV function of LFP is the thermodynamically-consistent OCV model shown in Figure 3(a) in the main manuscript, other parameters used in the simulation are parameterized by About:Energy Limited, initial SOC is set such that the cell has OCV of 3.65V when fully charged. Discharging time is set according to the corresponding experiments. The P2D simulation results matches the experimental results well except near the fully discharged region. The discrepancy between simulations and experiments may be resolved by fitting the LFP OCV to higher-quality OCV data.

prove this claim, we retrain the two models without the last 10 data points while keep other settings exactly the same. After the two models are fitted, they are applied to predict on the 10 unseen leave-out data points, all of which lie outside the fitted SOC region. Therefore, the two models are extrapolating when predicting on these 10 data points. Figure 3 shows the results, regular R-K model is non-monotonic when extrapolating and thus violates the

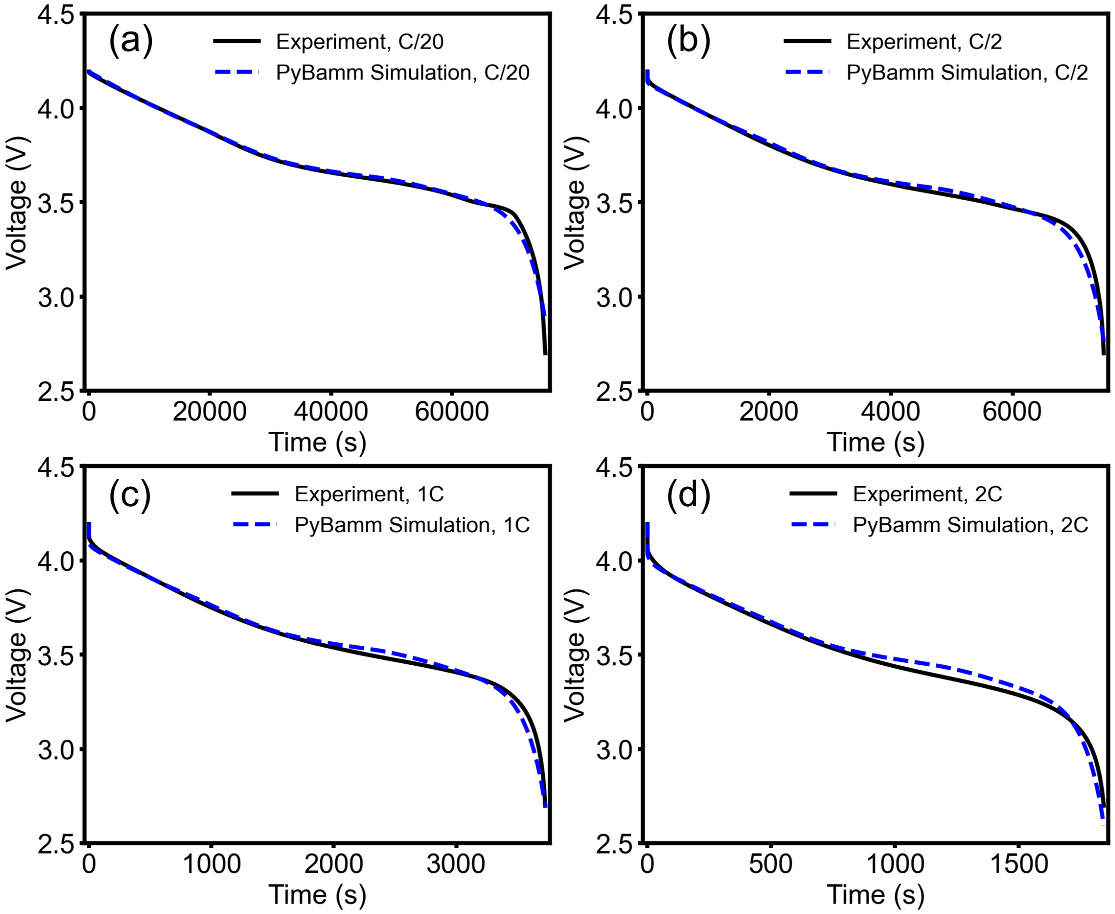

Figure 2: P2D discharge simulations (in blue dash lines) of a 12.5 Ah NMC111—Graphite Pouch Cell at room temperature at (a) C/20, (b) C/2, (c) 1C and (d) 2C, and the corresponding experimental results (in black solid line). For all the four simulations, the OCV function of graphite is the thermodynamically-consistent OCV model shown in Figure 3(j) in the main manuscript, other parameters used in the simulations are parameterized by About:Energy Limited, initial SOC is set such that the single cell in the pouch cell has OCV of 4.2V when fully charged. Discharging time is set according to the corresponding experiments. The P2D simulation results matches the experimental results well.

second law of thermodynamics, while thermodynamically consistent model obeys second law of thermodynamics. Furthermore, when SOC=0.96 which is within the extrapolation regime and outside the SOC range of LFP OCV dataset used in this work, the fitted regular R-K model predicts the OCV to be -13.51 V which is obviously unphysical, while the thermodynamically consistent model predicts 4.45 V. Scripts and results can be found at `https://github.com/BattModels/Diffthermo_OCV_paper/tree/main/LFP/extrapolation`

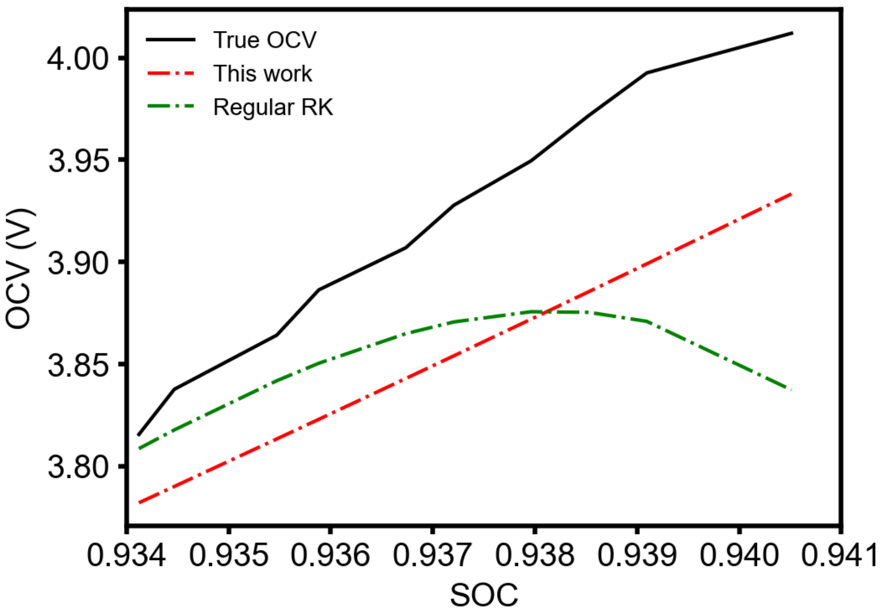

Figure 3: Experimental measured OCV values of the 10 leave-out data points, and the predicted OCV values by regular R-K model and thermodynamically-consistent model (referred to as this work)

Adding Splines At Boundaries of Miscibility Gaps

As mentioned in the main manuscript, the discontinuity of derivative of OCV with respect to SOC can be resolved by adding cubic splines at the boundaries of miscibility gaps. For a miscibility gap within SOC range of $x \in [x_\alpha, x_\beta]$, two splines, i.e. $OCV = a_{0\alpha} + a_{1\alpha}x + a_{2\alpha}x^2 + a_{3\alpha}x^3$ and $OCV = a_{0\beta} + a_{1\beta}x + a_{2\beta}x^2 + a_{3\beta}x^3$ can be constructed as alternative to the fitted OCV model within the SOC range of $[x_\alpha - \Delta x, x_\alpha + \Delta x]$ and $[x_\beta - \Delta x, x_\beta + \Delta x]$ respectively, such that the OCV value and the derivative of OCV with respect to SOC are continuous:

$$\begin{bmatrix} 1 & x_\alpha - \Delta x & (x_\alpha - \Delta x)^2 & (x_\alpha - \Delta x)^3 \\ 0 & 1 & 2(x_\alpha - \Delta x) & 3(x_\alpha - \Delta x)^2 \\ 1 & x_\alpha + \Delta x & (x_\alpha + \Delta x)^2 & (x_\alpha + \Delta x)^3 \\ 0 & 1 & 2(x_\alpha + \Delta x) & 3(x_\alpha + \Delta x)^2 \end{bmatrix} \begin{bmatrix} a_{0\alpha} \\ a_{1\alpha} \\ a_{2\alpha} \\ a_{3\alpha} \end{bmatrix} = \begin{bmatrix} U(x_\alpha - \Delta x) \\ U'(x_\alpha - \Delta x) \\ U(x_\alpha + \Delta x) \\ U'(x_\alpha + \Delta x) \end{bmatrix}$$

$$\begin{bmatrix} 1 & x_\beta - \Delta x & (x_\beta - \Delta x)^2 & (x_\beta - \Delta x)^3 \\ 0 & 1 & 2(x_\beta - \Delta x) & 3(x_\beta - \Delta x)^2 \\ 1 & x_\beta + \Delta x & (x_\beta + \Delta x)^2 & (x_\beta + \Delta x)^3 \\ 0 & 1 & 2(x_\beta + \Delta x) & 3(x_\beta + \Delta x)^2 \end{bmatrix} \begin{bmatrix} a_{0\beta} \\ a_{1\beta} \\ a_{2\beta} \\ a_{3\beta} \end{bmatrix} = \begin{bmatrix} U(x_\beta - \Delta x) \\ U'(x_\beta - \Delta x) \\ U(x_\beta + \Delta x) \\ U'(x_\beta + \Delta x) \end{bmatrix}$$

where $a_{0\alpha}, a_{1\alpha}, a_{2\alpha}, a_{3\alpha}$ are coefficients of the cubic spline fitted within the SOC range of $[x_\alpha - \Delta x, x_\alpha + \Delta x]$, Δx is a hyperparameter that decides the range of smoothed region, $U(x)$ is the OCV value given by the fitted thermodynamically consistent model at SOC=x, and $U'(x)$ is the $\frac{dOCV}{dSOC}$ value given by the fitted thermodynamically consistent model at SOC=x. Same notation applies to the other spline on the other side of miscibility gap. Since $U(x)$ and $U'(x)$ are decided by the R-K parameters and the solving process of the spline coefficients are implemented in PyTorch, the derivatives of spline coefficients with respect to R-K parameters can be also calculated by auto-differentiation, therefore keeping the whole OCV fitting program differentiable. A demonstration of this apporach can be found at `https://github.com/BattModels/Diffthermo_OCV_paper/blob/main/LFP/Discharge_4_RK_params_splines/train.py`. However, as mentioned in the main manuscript, this approach does not have thermodynamic basis. Future work might be developing other smoothing methods based on thermodynamics, but developing such methods are out of the scope of this work.

Comparison between Thermodynamically Consistent OCV Model and Monotonic R-K Model

Here we report the root mean square error (RMSE) of the OCV model proposed in this work and the monotonic R-K model on 12 different electrode materials shown in Figure 4 in the main text in Table 1.

Table 1: RMSE of the OCV model proposed in this work and the monotonic R-K model on 12 different electrode materials shown in Figure 4 in the main text.

Material	This Work (mV)	Monotonic R-K (mV)
LFP	14.49	117.40
LixFeSiO4	11.96	41.37
LCO	24.17	86.64
LMP	21.29	33.89
LMFP	45.17	230.38
LMO	12.10	46.16
NCO	10.27	22.96
NCA	6.28	3.47
NMC	6.13	7.52
Graphite	3.22	41.56
Si	5.30	14.26
LTO	35.36	165.61

OCV Models In Existing Literature That Violates Second Law of Thermodynamics

Besides 6 OCV models highlighted in the main text, Table 2 summarizes more OCV models in existing literature that violates the monotonic condition and therefore the second law of thermodynamics.

Table 2: Summary of some OCV models in existing literature that violates the monotonic condition, and thus the second law of thermodynamics.

Ref.	Description
[6]	R-K model for meso-carbon micro-beads, in Figure 3 as red solid line
[6]	2 Parameter Margules model for meso-carbon micro-beads, in Figure 3 as blue solid line
[7]	Skewed R-K model for LFP, on Page 125, Table 3.2.
[7]	Skewed R-K model for graphite, on Page 125, Table 3.2.
[7]	Skewed R-K model for LTO, on Page 125, Table 3.2.
[7]	Skewed R-K model for LCO, on Page 125, Table 3.2.

Continuation of Table 2	
Ref.	Description
[7]	Skewed R-K model for LMO, on Page 125, Table 3.2.
[7]	Skewed R-K model for NMC, on Page 125, Table 3.2.
[8]	OCV model for LFP, shown in Figure 1(a) as red solid line.
[9]	Model 2 shown in Figure 4.
[9]	Model 5 shown in Figure 4.
[10]	The 6-order polynomial OCV model shown in Figure 8.
[11]	The fourth-degree polynomial OCV models shown in Figure 2(a).
[11]	The fourth-degree polynomial OCV models shown in Figure 6(a).
[12]	Polynomial model shown in Figure 7(a)
[12]	Neural network model shown in Figure 8(c)
[13]	Model 1 shown in Figure 6
[13]	Model 2 shown in Figure 6
[13]	Model 3 shown in Figure 6
[13]	Model 4 shown in Figure 6
[13]	Model 5 shown in Figure 6
[14]	Neural network based OCV model at -25 $°C$ shown in Figure 10
[15]	Polynomial and logarithm OCV model shown in Figure 3(a) as blue line
[16]	10th-order Polynomial OCV model shown in Figure 5
[16]	15th-order Polynomial OCV model shown in Figure 8
[17]	Method 3 at -10 $°C$ shown in Figure 10
[17]	Method 5 at -10 $°C$ shown in Figure 14
[18]	Proposed $n = 2$ OCV model at 5 $°C$ and 15 $°C$ shown in Figure 8
[18]	Proposed $n = 3$ OCV model at 5 $°C$ and 15 $°C$ shown in Figure 8
[19]	Model 13 for OCV of LFP cells shown in Figure 3(d)

Continuation of Table 2	
Ref.	Description
[19]	Model 14 for OCV of LFP cells shown in Figure 3(d)
[19]	Model 15 for OCV of LFP cells shown in Figure 3(d)
[19]	Model 16 for OCV of LFP cells shown in Figure 3(d)
[20]	Model shown in Figure 5
[21]	Simplified electrochemical model shown in Figure 9(b)
[21]	Simplified electrochemical model shown in Figure 10(a)
[21]	Simplified electrochemical model shown in Figure 10(b)
[21]	Simplified electrochemical model shown in Figure 10(b)
[22]	Parametric-estimation OCV model at -20 $^{\circ}C$ shown in Figure 6
[23]	Simplified electrochemical model shown in Figure 7
[23]	Fourier model shown in Figure 7
[24]	PD-7 model shown in Figure 15
End of Table 2	

References

(1) Sulzer, V.; Marquis, S. G.; Timms, R.; Robinson, M.; Chapman, S. J. Python Battery Mathematical Modelling (PyBaMM). *Journal of Open Research Software* **2021**, *9*, 14.

(2) Marquis, S. G.; Sulzer, V.; Timms, R.; Please, C. P.; Chapman, S. J. An asymptotic derivation of a single particle model with electrolyte. *Journal of The Electrochemical Society* **2019**, *166*, A3693.

(3) About:Energy, About:Energy NMC111—Graphite & LFP—Graphite Cell Parameterisations for Battery Parameter eXchange (BPX). `https://github.com/About-Energy-OpenSource/About-Energy-BPX-Parameterisation`, 2023.

(4) Andersson, J. A. E.; Gillis, J.; Horn, G.; Rawlings, J. B.; Diehl, M. CasADi – A software framework for nonlinear optimization and optimal control. *Mathematical Programming Computation* **2019**, *11*, 1–36.

(5) Dreyer, W.; Jamnik, J.; Guhlke, C.; Huth, R.; Moškon, J.; Gaberšček, M. The thermodynamic origin of hysteresis in insertion batteries. *Nature materials* **2010**, *9*, 448–453.

(6) Karthikeyan, D. K.; Sikha, G.; White, R. E. Thermodynamic model development for lithium intercalation electrodes. *Journal of Power Sources* **2008**, *185*, 1398–1407.

(7) Plett, G. L. *Battery management systems, Volume I: Battery modeling*; Artech House, 2015; Vol. 1.

(8) Nejad, S.; Gladwin, D.; Stone, D. A systematic review of lumped-parameter equivalent circuit models for real-time estimation of lithium-ion battery states. *Journal of Power Sources* **2016**, *316*, 183–196.

(9) Weng, C.; Sun, J.; Peng, H. A unified open-circuit-voltage model of lithium-ion batteries for state-of-charge estimation and state-of-health monitoring. *Journal of power Sources* **2014**, *258*, 228–237.

(10) Pan, H.; Lü, Z.; Lin, W.; Li, J.; Chen, L. State of charge estimation of lithium-ion batteries using a grey extended Kalman filter and a novel open-circuit voltage model. *Energy* **2017**, *138*, 764–775.

(11) Yu, Q.; Wan, C.; Li, J.; E, L.; Zhang, X.; Huang, Y.; Liu, T. An open circuit voltage model fusion method for state of charge estimation of lithium-ion batteries. *Energies* **2021**, *14*, 1797.

(12) Dang, X.; Yan, L.; Jiang, H.; Wu, X.; Sun, H. Open-circuit voltage-based state of charge estimation of lithium-ion power battery by combining controlled auto-regressive and moving average modeling with feedforward-feedback compensation method. *International Journal of Electrical Power & Energy Systems* **2017**, *90*, 27–36.

(13) Zhang, Q.; Cui, N.; Li, Y.; Duan, B.; Zhang, C. Fractional calculus based modeling of open circuit voltage of lithium-ion batteries for electric vehicles. *Journal of Energy Storage* **2020**, *27*, 100945.

(14) Narayanan, S. S. S.; Thangavel, S. Machine learning-based model development for battery state of charge–open circuit voltage relationship using regression techniques. *Journal of Energy Storage* **2022**, *49*, 104098.

(15) Dong, G.; Wei, J.; Zhang, C.; Chen, Z. Online state of charge estimation and open circuit voltage hysteresis modeling of LiFePO4 battery using invariant imbedding method. *Applied Energy* **2016**, *162*, 163–171.

(16) Guo, Q.; White, R. E. Cubic spline regression for the open-circuit potential curves of a lithium-ion battery. *Journal of The Electrochemical Society* **2004**, *152*, A343.

(17) Gong, D.; Gao, Y.; Kou, Y. Parameter and state of charge estimation simultaneously for lithium-ion battery based on improved open circuit voltage estimation method. *Energy Technology* **2021**, *9*, 2100235.

(18) Chen, Y.; Yang, G.; Liu, X.; He, Z. A time-efficient and accurate open circuit voltage estimation method for lithium-ion batteries. *Energies* **2019**, *12*, 1803.

(19) Yu, Q.-Q.; Xiong, R.; Wang, L.-Y.; Lin, C. A comparative study on open circuit voltage models for lithium-ion batteries. *Chinese Journal of Mechanical Engineering* **2018**, *31*, 1–8.

(20) Song, Y.; Park, M.; Seo, M.; Kim, S. W. Improved SOC estimation of lithium-ion batteries with novel SOC-OCV curve estimation method using equivalent circuit model. 2019 4th International Conference on Smart and Sustainable Technologies (SpliTech). 2019; pp 1–6.

(21) Wang, L.; Sun, J.; Cai, Y.; Lian, Y.; Jin, M.; Zhao, X.; Wang, R.; Chen, L.; Chen, J. A novel OCV curve reconstruction and update method of lithium-ion batteries at different temperatures based on cloud data. *Energy* **2023**, *268*, 126773.

(22) Wang, Q.; Qi, W. New SOC estimation method under multi-temperature conditions based on parametric-estimation OCV. *Journal of Power Electronics* **2020**, *20*, 614–623.

(23) Liu, W.; Fu, S.; Li, Y.; Wan, B.; Wu, C.; Guo, R.; Pei, H.; Xie, J. A Study on State of Charge Estimation Method for Lithium Carbon Fluorides Primary Batteries. *Journal of The Electrochemical Society* **2022**, *169*, 120531.

(24) Abbas, M.; Cho, I.; Kim, J. Recursive multilayer perceptron-based data-driven identification for a parameterized polarization model of rechargeable Li-ion battery. *Applied Soft Computing* **2021**, *101*, 107073.
